# The Effect of Optically Induced Dielectrophoresis (ODEP)-Based Cell Manipulation in a Microfluidic System on the Properties of Biological Cells

**DOI:** 10.3390/bios10060065

**Published:** 2020-06-16

**Authors:** Po-Yu Chu, Chia-Hsun Hsieh, Chien-Ru Lin, Min-Hsien Wu

**Affiliations:** 1 Ph.D. Program in Biomedical Engineering, Chang Gung University, Taoyuan City 33302, Taiwan; l1a2yyy@gmail.com; 2 Division of Haematology/Oncology, Department of Internal Medicine, New Taipei Municipal Hospital, New Taipei City 23600, Taiwan; wisdom5000@gmail.com; 3 Division of Haematology/Oncology, Department of Internal Medicine, Chang Gung Memorial Hospital at Linkou, Taoyuan City 33302, Taiwan; 4 College of Medicine, Chang Gung University, Taoyuan City 33302, Taiwan; 5 Graduate Institute of Biomedical Engineering, Chang Gung University, Taoyuan City 33302, Taiwan; chianru0608@gmail.com; 6 Department of Chemical Engineering, Ming Chi University of Technology, New Taipei City 24301, Taiwan

**Keywords:** optically induced dielectrophoresis, microfluidic systems, cell manipulation, cell property, cell physiology

## Abstract

Cell manipulation using optically induced dielectrophoresis (ODEP) in microfluidic systems has attracted the interest of scientists due to its simplicity. Although this technique has been successfully demonstrated for various applications, one fundamental issue has to be addressed—Whether, the ODEP field affects the native properties of cells. To address this issue, we explored the effect of ODEP electrical conditions on cellular properties. Within the experimental conditions tested, the ODEP-based cell manipulation with the largest velocity occurred at 10 Vpp and 1 MHz, for the two cancer cell types explored. Under this operating condition, however, the cell viability of cancer cells was significantly affected (e.g., 70.5 ± 10.0% and 50.6 ± 9.2% reduction for the PC-3 and SK-BR-3 cancer cells, respectively). Conversely, the exposure of cancer cells to the ODEP electrical conditions of 7–10 Vpp and 3–5 MHz did not significantly alter the cell viability, cell metabolic activity, and the *EpCAM*, *VIM*, and *ABCC1* gene expression of cancer cells. Overall, this study fundamentally investigated the effect of ODEP electrical conditions on the cellular properties of cancer cells. The information obtained is crucially important for the utilization of ODEP-based cell manipulation in a microscale system for various applications.

## 1. Introduction

The fine manipulation of biological cells has attracted considerable interests in a wide variety of biomedical studies (e.g., cell sorting [1,2,3,4,5,6,7,8], the isolation and purification of cells [1,2,3,4,5,6,7,8,9], cell patterning [10,11,12,13,14], tissue engineering [10,11,14], or various cell-based bioassays [12,13]). These attempts are technically challenging to realize conventionally, due to the inadequate scale of tools or equipment used for cell manipulation in general biological laboratories. Leveraging the appropriate dimensional feature in a microfluidic system, and various novel approaches for microparticle manipulations (e.g., acoustophoresis [1,13], magnetophoresis [2], thermophoresis [15], and dielectrophoresis (DEP) [3,4]), researchers could achieve the above-mentioned goals in a high performance manner [16,17].

Among the techniques for microparticle manipulation in a microfluidic system, microparticle manipulation using DEP mechanism has been widely adopted for various applications (e.g., microparticle enrichment [18,19], microparticle patterning [10,11], cell-type classification [20], or rare cell isolation [3,4]). DEP-based microparticle manipulation, first presented in the 1950s [21], is well-described elsewhere [22], and can be briefly described as follows. When dielectric microparticles (e.g., biological cells) are suspended in a solution in which an electric field is exerted, charges can be electrically polarized on the microparticles’ surface. The interaction between the induced charges on the microparticles and the electric field specifically exerted on them can generate a DEP force on such microparticles [22]. In practice, therefore, the scientists can delicately control the electric field exerted on biological cells via a designed microelectrode array, to manipulate these cells in a manageable manner. Although the DEP technique is proven feasible for the fine manipulation of cells, this technique normally requires a costly, time-consuming, and technically demanding microfabrication process to create a unique metal microelectrode layout that is specific to the application [23,24]. In addition, cell manipulation using the DEP mechanism might be technically challenging for general scientists, due to its complexity. To tackle the technical issues, the optically induced dielectrophoresis (ODEP)-based technique could open up a new horizon for the manipulation of microparticles in a more efficient, flexible, and user-friendly manner [23,24].

Microparticle manipulation based on the ODEP mechanism was first proposed in 2005 [23]. Similar to the DEP mechanism described earlier, an electric voltage is applied between the top and bottom substrates of an ODEP system to generate a uniform electric field in the solution layer sandwiched between the two substrates. Under this circumstance, the dielectric microparticles suspended in the solution layer are electrically polarized. In contrast to the DEP mechanism, however, the key technical merit of the ODEP mechanism is that it can easily and quickly create or modify an electrode layout through the control of optical patterns, by acting as a virtual electrode [23,24]. When the photoconductive layer of an ODEP system is briefly projected with light, it can lead to a significant reduction in electrical impedance in the light-illuminated area. This phenomenon can cause the exerted voltage to decrease across the solution layer inside the light-projected region. This phenomenon, therefore, generates a locally nonuniform electric field within an ODEP system. For microparticle manipulation, scientists can simply utilize a commercial projector to project optical images on an ODEP system, by which the interaction between the generated nonuniform electric field and an electrically polarized microparticle is used to manipulate the microparticle. In terms of operation, one can control the light image projected onto the ODEP system and thus the nonuniform electric field created to manipulate dielectric microparticles within the ODEP system.

The use of ODEP mechanism in microfluidic systems has been successfully applied for the high-accuracy positioning and assembling of metallic beads [25], dynamic analysis of cancer-immune microenvironment [26], or the isolation and purification of rare cell species in clinical samples (e.g., Raji cells [7], circulating tumor cells (CTCs) [5,9], and CTC clusters [6]) in a higher performance manner than their conventional counterparts. Although the incorporation of the ODEP mechanism in a microscale system has provided a powerful tool for the biological cell-relevant studies or applications, one fundamental question has to be answered before its widespread applications—Whether the cell manipulation using the ODEP mechanism influences the cellular properties. If this is the case, the use of such a mechanism for cell manipulation could complicate the subsequent biological studies or applications. To the best of our knowledge, however, this fundamental issue has not yet been well explored.

To investigate the effect of ODEP-based cell manipulation (the operating conditions explored—(1) the magnitude of AC voltage: 5–10 V; (2) frequency of AC voltage: 1–5 MHz, (3) operating time: 3 min) on the properties (e.g., cell viability, metabolic activity of cells, or gene expression of cells) of biological cells (e.g., PC-3 and SK-BR-3 cancer cells), we performed various experiments. Within the experimental conditions investigated, the ODEP-based cell manipulation with the best performance (e.g., highest cell manipulation velocity) occurred at 10 Vpp and 1 MHz, for the two cancer cell types explored. Under this ODEP electrical condition, however, the cell viability of the cancer cells tested was significantly affected (e.g., 70.5 ± 10.0% and 50.6 ± 9.2% reduction for the PC-3 and SK-BR-3 cancer cells, respectively), possibly due to the electrical lysis of cells. Conversely, the exposure of cancer cells to the ODEP field with particular conditions (e.g., 7–10 Vpp, 3–5 MHz, and 3 min exposure time) might not significantly alter the cell viability, cell metabolic activity, and gene expression of cancer cells. Within this ODEP operating condition, the highest maximum velocity (e.g., 106.7 ± 30.6 μm s^−1^ and 100.0 ± 20.0 μm s^−1^ for the PC-3 and SK-BR-3 cancer cells, respectively) of a light image that can manipulate cells occurred at the voltage magnitude and frequency of 10 Vpp and 3 MHz, respectively. The above-mentioned cell manipulation velocities are technically sufficient for various applications. As a whole, this study fundamentally investigated the effect of ODEP electrical conditions on the cellular properties of cancer cells. The information obtained is crucially important for the utilization of ODEP-based cell manipulation in a micro-scale system for various applications.

## 2. Materials and Methods

### 2.1. Microfluidic Chip and Experimental Setup

**Figure 1:**
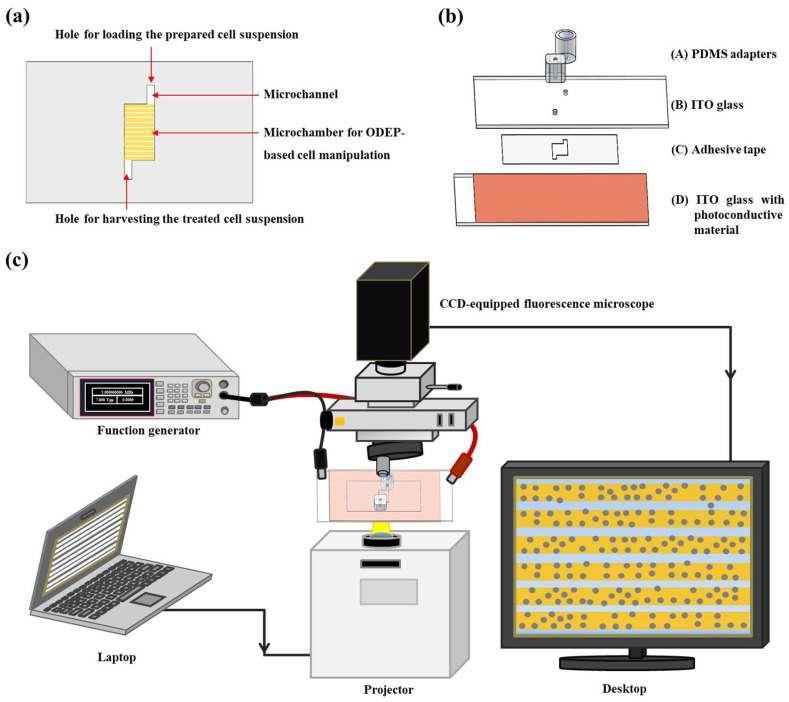
Schematic presentation of (**a**) the design of the microfluidic chip (top view), (**b**) the structure of the microfluidic chip ((A) polydimethylsiloxane (PDMS) adapters for tubing connection, (B) indium-tin oxide (ITO) glass, (C) processed adhesive tape, and (D) ITO glass with a coating layer of photoconductive material), and (**c**) the overall experimental setup.

To explore the effect of ODEP on the properties of biological cells, we designed a simple microfluidic chip. Figure 1a schematically presents the top-view layout of the microfluidic chip, mainly encompassing a microchamber (L = 6.0 mm, W = 4.0 mm, and H = 50.0 µm) connecting two microchannels (L = 2.0 mm, W = 1.0 mm, and H = 50.0 µm). In this work, an ODEP field was applied to the microchamber (Figure 1a). The structure of the microfluidic chip is illustrated in Figure 1b. Briefly, the microfluidic chip consists of two custom-made polydimethylsiloxane (PDMS) (Sylgard^®^ 184, Dow Corning, Midland, MI, USA) adapters for tubing connection (A), an indium-tin oxide (ITO) glass (10 Ω, 0.7 mm; Innolux Corp., Miaoli County, Taiwan) (B), a processed adhesive tape (L298, Sun-yieh, New Taipei City, Taiwan; thickness: 50 µm) containing hollow microchamber and microchannel structures (C), and a bottom ITO glass (D) with a coating layer of photoconductive material (a 1.0 μm-thick hydrogenated amorphous silicon (a-Si:H) layer). The fabrication processes (e.g., PDMS replica mold, metal mold-punching fabrication, plasma-enhanced chemical vapor deposition (PECVD)-based thin-film technology, and plasma oxidation-aided bonding) were well-described previously [8,27,28]. After each substrate was fabricated (Figure 1b), substrate A was bonded with substrate B through the surface treatment of plasma oxidation. This step was followed by the assembly with the substrate D with the aid of the processed double-sided adhesive tape (substrate C) (Figure 1b). In operation, the prepared cell suspension was manually loaded into the microchamber, using a pipette and a tip. For cell manipulation using the ODEP mechanism, the commercially available ODEP-based cell isolation equipment (Celnostics, Ace Medical Technology Co., Ltd., Taipei City, Taiwan) was used to achieve the ODEP-based cell manipulation in the proposed microfluidic chip. Within this equipment, briefly, a function generator (AFG-2125, Good Will Instrument Co., Ltd., New Taipei City, Taiwan) was utilized to apply an AC bias between the two ITO glasses. A computer-interfaced digital projector (EB-X05, Epson, Nagano, Japan) was used to illuminate light images onto the photoconductive material (i.e., the Substrate D) of microfluidic chip to generate the ODEP force on the cells. An illustration of the important operating modules of the equipment is schematically presented in Figure 1c (a photograph of the overall experimental setup is provided in a Supplementary Figure S1).

### 2.2. The Assessment of the ODEP Manipulation Force Generated on Cells

The working principle of ODEP for cell manipulation is described in the Introduction. The ODEP force generated on a cell can theoretically be described by Equation (1) [24,27,28]:

*F_DEP_* = 2π*r*^3^*ε_0_ε_m_*Re[f_CM_]*∇|E|*^2^ (1)

In Equation (1), *r* (cellular radius), *ε_0_* (vacuum permittivity), *ε_m_* (relative permittivity of the surrounding solution), *∇|E|^2^* (gradient of electric field squared), and Re[f_CM_] (real part of the Clausius–Mossotti factor (f_CM_)) are the important parameters [24,28]. The f_CM_ is described by Equation (2) [29,30,31]:

(2) $$f_{\mathrm{CM}}=\frac{\varepsilon_{cell}^{*}-\varepsilon_{m}^{*}}{\varepsilon_{cell}^{*}+2\varepsilon_{m}^{*}}$$

where $\varepsilon_{cell}^{*}$ and $\varepsilon_{m}^{*}$ represent the complex permittivity of the cell and the surrounding solution, respectively. For a single-cell model, the complex permittivity of the cell and the surrounding solution can be further described by Equations (3) and (4):

(3) $$\varepsilon_{cell}^{*}=C_{mem}^{*}\frac{3R\varepsilon_{int}^{*}}{3\varepsilon_{int}^{*}+3C_{mem}^{*}R},C_{mem}^{*}=\frac{\varepsilon_{mem}}{d}-\frac{j\sigma_{mem}}{d},\varepsilon_{int}^{*}=\varepsilon_{0}\varepsilon_{int}-\frac{j\sigma_{int}}{\omega }$$

(4) $$\varepsilon_{m}^{*}=\varepsilon_{0}\varepsilon_{m}-\frac{j\sigma_{m}}{\omega }$$

In Equations (3) and (4), $C_{mem}^{*}$ represents the complex cell membrane capacitance, $\varepsilon_{int}^{*}$ represents the complex permittivity of the cellular interior (i.e., cell cytoplasm), *R* represents the radius of the cellular interior, d represents the thickness of cell membrane, ε represents the relative permittivity of the cell membrane, cellular interior, or surrounding solution (denoted by the subscript *mem*, *int*, or *m*, respectively), σ represents the conductivity of the cell membrane, cell interior, or surrounding solution (denoted by the subscript *mem*, *int*, or *m*, respectively), j represents the imaginary vector ($\sqrt{-1}$), and ω represents the angular frequency (i.e., *ω* = 2π*f*) of the applied AC field, respectively [29,30,31]. Under a given solution condition, overall, the magnitude and frequency of the electric voltage applied could play important roles in the ODEP force generated on a particular cell [24,28]. In this work, the effect of electric conditions (i.e., the magnitude and frequency of the electric voltage exerted) on the ODEP force generated on cells was experimentally evaluated. In the evaluation, the ODEP manipulation force, a net force between the ODEP and the friction force, acting on the manipulated cells was then experimentally assessed, based on the method described previously [8,27,28]. In a steady state, the ODEP manipulation force acting on a cell was balanced by the viscous drag of fluid acting on such a cell under a continuous flow condition. As a result, the hydrodynamic drag force of a moving cell was used to evaluate the net ODEP manipulation force of a cell according to Stokes’ law (Equation (4)):

*F* = 6π*rηv* (5)

In Equation (5), *r* (cellular radius), *η* (fluidic viscosity), and *v* (the velocity of a moving cell) are the important parameters. Under the given solution and cellular size conditions, overall, the ODEP manipulation force of the manipulated cell could then be experimentally assessed through the measurement of the maximum velocity of a moving optical image that can manipulate such a cell [8,27,28]. In practice, briefly, a light bar image with different moving velocities (e.g., from low to high velocities) was used to manipulate a cell (e.g., attracted and pulled a cell). Through this process, the maximum velocity of a moving optical image that can manipulate such a cell was then determined. In this work, therefore, the above-mentioned velocity was utilized as an index for the evaluation of the ODEP manipulation force generated on a specific cell under a particular electric condition. Based on this, the effect of electric conditions (e.g., magnitude of AC electric voltage: 7–10 Vpp and frequency of AC electric voltage: 1–5 MHz) on the ODEP manipulation of the cells tested (e.g., PC-3 and SK-BR-3 cancer cells) was evaluated. Briefly, the cell sample tested was prepared in a cell suspension (cell density: 10^6^ cells mL^−1^), followed by loading into the microchamber of the microfluidic chip (Figure 1a). The maximum velocity of a moving light bar (L: 1.3 mm W: 100.0 μm) that could manipulate these cells was then assessed [27,28].

### 2.3. Evaluation of the Properties of Cancer Cells Treated with Varied ODEP Operating Conditions

For the analysis of the ODEP effect on the cellular properties, the cancer cells tested (e.g., PC-3 and SK-BR-3 cancer cell lines, two of the commonly-used cancer cell lines in cancer-related studies [32,33]) were first treated with the ODEP fields under different conditions for 3 min, followed by assaying their cellular properties, including cellular viability, cellular metabolism activity, and gene expression. In this study, the biological assays were carried out at 1.5 ± 0.2 h after the ODEP exposure treatment. In brief, the background medium of the prepared cancer cell suspension (cell density: 5 × 10^6^ cells mL^−1^ for PC-3 cancer cells, and 3 × 10^6^ cells mL^−1^ for SK-BR-3 cancer cells) was first replaced by a 9.5% (*w/v*) sucrose buffer solution (relative permittivity: ~76.19 [34], fluid viscosity: ~1.0389 × 10^−2^ g s^−1^ cm^−1^ [35], osmolality: 270–290 mOsmol kg^−1^, and conductivity: 1–5 μS cm^−1^), the commonly used low conductivity buffer solution for ODEP-based cell manipulation [5,8,9,27,28]. The processed cell suspension sample was then loaded into the microchamber of a microfluidic chip (Figure 1a). In this process, however, a small portion of cells might retain in the microchannel area. In this work, the cell manipulation using ODEP was first performed to quickly (operation time—within 5 s) transport these cells to the microchamber area before ODEP treatment. At the microchamber zone, 40 rectangular light bar images (L: 4.0 mm, W: 100.0 μm, the interval between light bars: 50.0 μm) were then illuminated to provide an ODEP field on the cancer cells tested. In this study, ODEP fields with different electric conditions (magnitude of AC electric voltage: 7–10 Vpp and frequency of AC electric voltage: 1–5 MHz) were used to treat the cancer cells tested. After exposure to the ODEP field for 3 min, the treated cells were then harvested for the subsequent bioassays. In the process, briefly, a suction-type syringe pump was utilized to collect the treated cancer cells through the hole for harvesting cells (Figure 1a). Before the following bioassays, the collected cells were kept in the form of cell suspension in the original sucrose buffer solution under the thermal condition of 25 °C.

In this work, the commonly-used Cell Counting Kit-8 (CCK-8) (CK04–05, Dojindo, Kumamoto, Japan) and ATP Colorimetric/Fluorometric assays (K354-100, BioVision Inc., Milpitas, CA, USA) were utilized to assay the cell viability and cell metabolic activity of cancer cells, respectively. The bioassays were carried out according to the manufacturer’s instructions. For further analysis of whether cell manipulation using ODEP could affect cellular gene expression, the expression levels of genes in cancer cells treated with different ODEP fields were experimentally evaluated. As the ODEP-based cell manipulation was commonly used for the isolation and purification of circulating tumor cells (CTCs) [5,9], the important CTC-related gene expressions were thus explored in this work. For the cancer cells (PC-3 and SK-BR-3 cancer cells) tested, the mRNA levels of epithelial-to-mesenchymal transition (EMT)-associated genes [*EpCAM* (Hs00158980_m1) and *VIM* (Hs00958111_m1)], the multidrug resistance-associated protein 1 (MRP1) gene [*ABCC1* (Hs01561502_m1)], and the housekeeping gene [*GAPDH* (Hs02758991_g1)] were experimentally quantified. The bioassay was based on a method described previously [8,9,27]. In brief, RNA was extracted from the cancer cells tested using a bromochloropropane (BCP)-based TRI Reagent procedure (Thermo Fisher Scientific, San Jose, CA, USA [36]). This process was followed by the reverse transcription using a SuperScript^®^ IV Reverse Transcriptase Kit (Thermo Fisher Scientific, San Jose, CA, USA). The mRNA level was subsequently quantified using a StepOne™ Real-Time PCR System (Thermo Fisher Scientific, San Jose, CA, USA).

### 2.4. Statistical Analysis

In this study, data were obtained from three separate experiments, and are presented as the mean ± standard deviation (n = 9). To compare the results from different operating conditions, we used one-way ANOVA and Tukey’s honestly significant difference (HSD) post-hoc test for the statistical analysis.

## 3. Results and Discussion

### 3.1. Effect of the Electric Conditions on ODEP-Based Cell Manipulation

**Figure 2:**
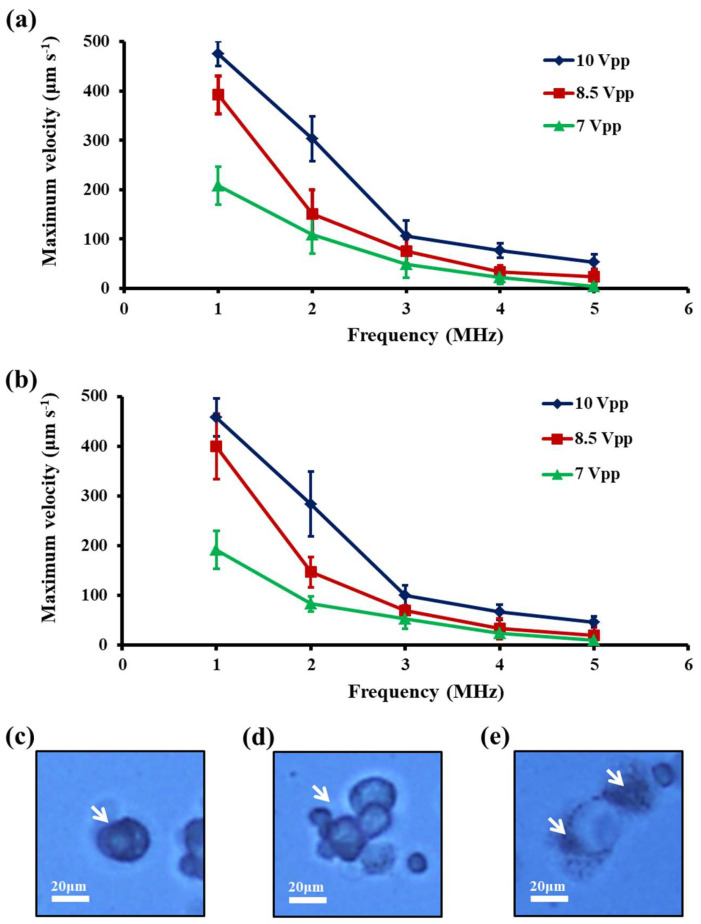
The quantitative relationship between the maximum velocity of a moving light bar that could manipulate the (**a**) PC-3 and (**b**) SK-BR-3 cancer cells tested and the frequency (1, 2, 3, 4, and 5 MHz) of the electric voltage applied under different voltage magnitudes (7, 8.5, and 10 Vpp) conditions and bright-field microscopic images showing the phenomena of (**c**) cell adhesion (10 Vpp and 500 kHz), (**d**) cell aggregation (10 Vpp and 750 kHz), and (**e**) cell lysis (10 Vpp and 100 kHz), during the exposure of cancer cells to the ODEP field with a voltage magnitude and frequency, as indicated.

In this study, the effect of the ODEP electrical conditions (e.g., magnitude of AC electric voltage: 7–10 Vpp, and frequency of AC electric voltage: 1–5 MHz) on the cell manipulation of the cancer cells (e.g., PC-3 and SK-BR-3 cancer cells) tested were experimentally evaluated. Figure 2 reveals the quantitative relationship between the maximum velocity of a moving light bar that can manipulate the cancer cells tested (thus, the ODEP manipulation force of the cancer cells; Equation (5)) and the frequency of electric voltage applied under different voltage magnitude conditions. The results (Figure 2a,b) showed that the highest values of the maximum velocity of a light image that can manipulate the cancer cells, both occurred at 10 Vpp and 1 MHz for the two cancer cell types explored (i.e., 475.0 ± 25.0 μm s^−1^ and 458.3 ± 38.2 μm s^−1^ for the PC-3 and SK-BR-3 cancer cells, respectively). When the voltage frequency was lower than 1 MHz, some undesirable phenomena such as cell adhesion (10 Vpp and 500 kHz) (Figure 2c), cell aggregation (10 Vpp and 750 kHz) (Figure 2d), and cell lysis (10 Vpp and 100 kHz) (Figure 2e), would occur that could significantly affect the cellular properties [28]. Within the experimental conditions explored, moreover, the appearance rate of the above-mentioned phenomena was near 100% when the operating conditions were set at the particular electric conditions as indicated. These resulting phenomena might be due to the enhancement of electrically induced cell deformability, mutual DEP, and electrical lysis of cells at a voltage frequency near or below 1 MHz [26]. When the electric condition was set at 7 Vpp and 5 MHz (Figure 2a,b), conversely, we observed that the maximum velocities of a light image that can manipulate the cancer cells tested both reached their lowest levels for the two cancer cell types tested (i.e., 3.3 ± 5.8 μm s^−1^ and 10.0 ± 10.0 μm s^−1^ for the PC-3 and SK-BR-3 cancer cells, respectively) within the experimental conditions investigated. When the voltage magnitude and frequency were lower and higher than 7 Vpp and 5 MHz, respectively, the above-mentioned velocities were further decreased, which might significantly affect cell manipulation using the ODEP mechanism. Based on the facts described above, the optimal window of electric conditions for effective cell manipulation using ODEP was set at 7–10 Vpp and 1–5 MHz for the voltage magnitude and frequency, respectively. The ODEP operating conditions within such ranges were also reported for the ODEP-based cell manipulation for different applications [7,8,27,28]. Within this electric condition range, moreover, the maximum velocity of a light image that could manipulate the cancer cells tested decreased significantly with an increase of voltage frequency (Figure 2a,b), which was also consistent with previous findings [28]. Furthermore, under a given voltage frequency condition, the increase in voltage magnitude was found to increase the maximum velocity of a light image that could manipulate the cancer cells tested. This phenomenon could be explained by Equation (1), in which the ODEP force generated on a cell is proportional to the electric field squared. In addition to the values of the maximum velocity of a light image (and thus the ODEP force) investigated in the above-mentioned studies, it might also be valuable to explore the values of electric-field distribution in ODEP-based cell manipulation.

### 3.2. Effect of the ODEP Field on the Cellular Viability of Cancer Cells

**Figure 3:**
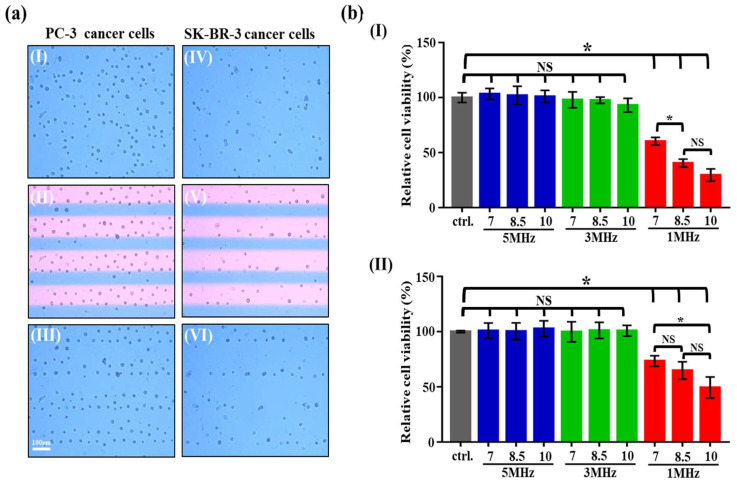
(**a**) Microscopic observation (close-up view) of the cancer cells in the microchamber of the microfluidic chip before (the upper row), during (the middle row), and after (the lower row) treatment with ODEP exposure (the left column—PC-3 cancer cells; the right column—SK-BR-3 cancer cells), (**b**) the comparison of relative cell viability (%) of (I) the PC-3 cancer cells and (II) the SK-BR-3 cancer cells treated with the ODEP field with varied electric conditions (voltage magnitude and frequency: 7, 8.5, and 10 Vpp and 1, 3, and 5 MHz, respectively) (NS—No significant difference (*p* > 0.05), * significant difference (*p* < 0.05)).

In this study, PC-3 and SK-BR-3 cancer cells, two of the commonly used cancer cell lines in the cancer-related studies, were used as model cells to investigate the effect of the ODEP-based cell manipulation on cellular properties. In the operations, the prepared cancer cell suspension was loaded into the microchamber of the ODEP microfluidic chip (Figure 1a), followed by exposure to the ODEP field with varied electric conditions (i.e., 7–10 Vpp and 1–5 MHz for the voltage magnitude and frequency, respectively), as determined previously (Figure 2). In our preliminary tests, the impact of ODEP exposure time on the cell viability of PC-3 cancer cells was investigated. Results (Supplementary Figure S2) showed that the cell viability of such cells had no significant difference (*p* > 0.05) compared to the control (i.e., the cancer cells without exposure to the ODEP field) within the 5 min exposure time, when the voltage magnitude and frequency were set at 10 Vpp and 5 MHz, respectively. When the ODEP exposure time was as long as 10 and 15 min, conversely, the cell viability of the PC-3 cancer cells might be significantly affected (*p* < 0.05) (e.g., 43.7 ± 5.5% and 51.3 ± 10.3% reduction for the ODEP exposure time of 10 and 15 min, respectively) in comparison to the control. Based on the preliminary results, therefore, the ODEP exposure time was set at 3 min, and within the time period, general cell manipulation using the ODEP mechanism was normally carried out (e.g., the rapid separation of Raji cells [7] or isolation and purification of CTCs [5,9]). In practice, 40 rectangular light bar images were illuminated on the microchamber zone. During the period of ODEP exposure, the cancer cells tested were attracted within the light bars, as shown in Figure 3(aII,aV). After the exposure to the ODEP field, we observed microscopically that the treated cancer cells within the microchamber were aligned in accordance with the original light bar images, as exhibited in Figure 3 (aIII,aVI). Then, the ODEP-treated cancer cells were collected for the following bioassays.

In this study, the Cell Counting Kit-8 (CCK-8) was utilized to investigate the viability of cancer cells treated with various ODEP fields. The results (Figure 3(bI)) revealed that the cell viability of PC-3 cancer cells had no significant difference (*p* > 0.05) compared to that of the control cells (i.e., the cancer cells without exposure to the ODEP field), when the voltage magnitude and frequency were set at 7–10 Vpp and 3–5 MHz, respectively. A similar result was also found for the SK-BR-3 cancer cells tested (Figure 3(bII)). However, once the voltage frequency was set at 1 MHz, the cell viability of the PC-3 and SK-BR-3 cancer cells decreased significantly (*p* < 0.05) compared to that of the control, irrespective of a voltage magnitude of 7, 8.5, or 10 Vpp. This finding could be explained as follows. If the equivalent circuit of a biological cell is assumed to be a lumped circuit, the electrical property of the cell membrane will be similar to the electric capacitance [28]. When the voltage frequency is altered from high to low frequency (e.g., from 10 MHz to 100 kHz), the electrical impedance of a cell membrane might increase. This phenomenon could, therefore, result in the shift of the exerted electric voltage from the cell cytoplasm to the cell membrane of a cell [28]. This fact might in turn lead to an increase in the transmembrane potential in a cell membrane when the voltage frequency is changed from high to low frequency. The above-mentioned phenomena could explain the low cell viability caused by cell electrical lysis (Figure 2e) when the voltage frequency is set at low conditions (e.g., 1 MHz) (Figure 3b).

Under the low voltage frequency condition of 1 MHz (Figure 3(bI)), moreover, the cell viability of the PC-3 cancer cells significantly decreased (*p* < 0.05) when the magnitude of voltage was higher than 8.5 Vpp (observed cell viability: 40.4 ± 3.6%). A similar result was also found for the SK-BR-3 cancer cells tested (Figure 3(bII)), in which the cell viability of the SK-BR-3 cancer cells was significantly downregulated (*p* < 0.05) (the observed cell viability: 49.4 ± 9.5%) when the magnitude of the voltage was higher than 10 Vpp. Overall, the lower cell viability of cancer cells found at the higher voltage condition could be explained by the fact that the electrical lysis of cells is prone to occur when a cell is exposed to a high-voltage magnitude [37,38]. Taken together, the increase of voltage magnitude or decrease of voltage frequency, respectively, could raise the maximum velocity of a light image that can manipulate cells (i.e., the ODEP manipulation force of cells; Equation (5), Figure 2). Nevertheless, the increase in voltage magnitude or decrease in voltage frequency, respectively, could accordingly increase the tendency of electrical lysis of cells, which could affect cellular viability. Within the experimental conditions explored (i.e., 7–10 Vpp and 1–5 MHz), the determinant electrical condition that could significantly affect cell viability was a low-voltage frequency of 1 MHz, when the voltage magnitude range was 7–10 Vpp.

### 3.3. Effect of the ODEP Field on the Cellular Metabolic Activity and Gene Expression of the Cancer Cells Tested

**Figure 4:**
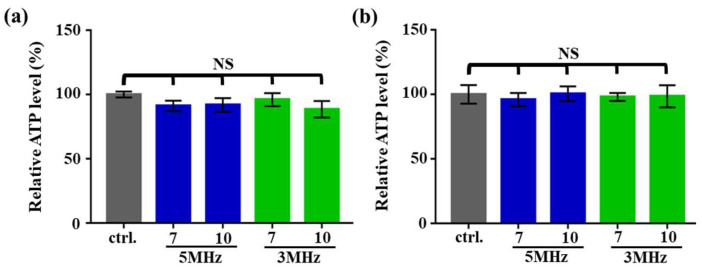
Comparison of the relative ATP levels (%) of (**a**) the PC-3 cancer cells and (**b**) the SK-BR-3 cancer cells treated with the optically induced dielectrophoresis (ODEP) field under varied electric conditions (voltage magnitude and frequency—7 and 10 Vpp and 3 and 5 MHz, respectively) [NS—No significant difference (*p* > 0.05)].

As suggested by the previous results (Figure 3b), cell manipulation using the ODEP mechanism at a low voltage frequency of 1 MHz could be lethal to the cancer cells tested. Such an ODEP electrical condition was ruled out in subsequent studies. In the following evaluations, the effect of the ODEP fields with varied electrical conditions (i.e., voltage magnitude and frequency: 7–10 Vpp and 3–5 MHz, respectively) on the metabolic activity and gene expression of cancer cells were explored. For the former, the ATP synthesis of cells was used as an indicator based on the previous studies [39]. The results (Figure 4a) showed that the ATP level of the ODEP-treated PC-3 cancer cells showed no significant difference (*p* > 0.05) compared to that of the control (i.e., the cancer cells without exposure to the ODEP field), within the experimental conditions explored. A similar result was also observed for the SK-BR-3 cancer cells (Figure 4b). The findings in this study could indicate that the ODEP field could not affect the ATP synthesis of a cell within the electric conditions tested. This result was, to some extent, similar to that found in a previous DEP-based study [40].

**Figure 5:**
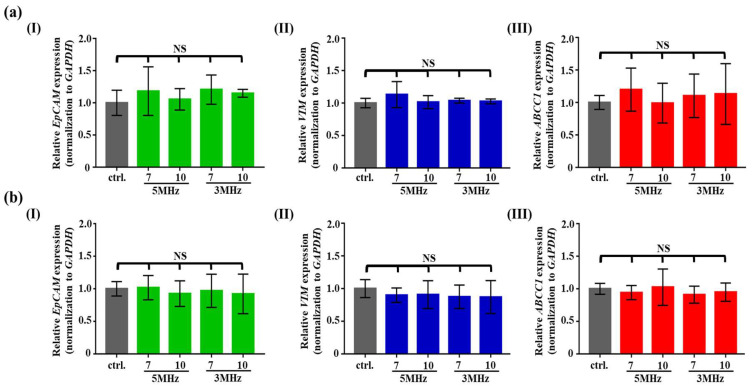
The comparison of relative gene expression ((I) *EpCAM*, (II) *VIM*, and (III) *ABCC1* genes) of (**a**) the PC-3 cancer cells and (**b**) the SK-BR-3 cancer cells treated with the ODEP field with varied electrical conditions (voltage magnitude and frequency: 7 and 10 Vpp and 3 and 5 MHz, respectively) (NS—No significant difference (*p* > 0.05).

In addition to the effect on cellular metabolic activity, the ODEP effect on the gene expression of cancer cells was investigated. In this study, the gene expression of EMT-associated genes (e.g., *EpCAM* and *VIM*) [8] and the MRP1 gene (e.g., *ABCC1*) [8] in the PC-3 and SK-BR-3 cancer cells was quantified and then normalized to the gene expression of *GAPDH*. The results (Figure 5a) showed that the gene expression of the above-mentioned genes in the PC-3 cancer cells was not significantly different (*p* > 0.05) from that of the control cells (i.e., the cancer cells without exposure to the ODEP field) within the ODEP operating conditions (i.e., 7–10 Vpp, 3–5 MHz, and 3 min exposure time) studied. A similar outcome (Figure 5b) was also found for the SK-BR-3 cancer cells.

Previous studies similar to this work revealed that the exposure to a DEP field (e.g., 20 Vpp, 250 kHz, and 1 h exposure time) could downregulate the expression of cell differentiation-related genes (e.g., *ADGRE5/CD97*, *RUNX2*, and *NES*) in mesenchymal stem cells (e.g., UE7T-13 cells) [41]. It was also reported that the exposure to a DEP field (e.g., 21 Vpp, 5 MHz, and 15 min exposure time) might not affect the cell morphology, cell oxidative respiration rate, and cell cycle dynamics of fibroblast-like cells (e.g., BHK-21/C13 cells) [40]. After exposure to the DEP field, however, the gene expression of the c-fos protein (commonly activated by environmental stress [42]) was observed to be upregulated [40]. The reasons behind the discrepancies found in the previous and current studies might be complicated and could be related to the cell species, electrical conditions, and the genes explored. Further experiments will be required to systematically investigate this issue. In addition to the gene expressions investigated in this study, it might also be valuable to explore the expression profiles of the genes regulating the signal pathways downstream of voltage-gated ion channels.

## 4. Conclusions

The ODEP-based microsystem provides a powerful tool for biological cell-relevant studies or applications. Before its widespread application, one fundamental issue has to be addressed—Whether cell manipulation using the ODEP mechanism influences the native properties of cells. To address this issue, we carried out various experiments. In this study, the impact of the ODEP electrical conditions ((1) the magnitude of AC voltage: 5–10 V; (2) frequency of AC voltage: 1–5 MHz), (3) operating time: 3 min) on the properties (e.g., cell viability, metabolic activity of cells, or gene expression of cells) of biological cells (e.g., PC-3 and SK-BR-3 cancer cells) was explored. Within the experimental conditions studied, the ODEP-based cell manipulation with the highest performance (e.g., highest cell manipulation velocity) occurred at 10 Vpp and 1 MHz for the two cancer cell types explored. Under this ODEP electrical condition, however, the cell viability of the cancer cells tested was significantly affected (e.g., 70.5 ± 10.0% and 50.6 ± 9.2% reduction for the PC-3 and SK-BR-3 cancer cells, respectively) possibly due to cell electrical lysis. Conversely, the exposure of cancer cells to the ODEP field with a particular condition range (i.e., 7–10 Vpp, 3–5 MHz, and 3 min exposure time) might not significantly alter the cell viability, cell metabolic activity, and the gene expression of cancer cells. Within this ODEP operating condition, the highest maximum velocity (i.e., 106.7 ± 30.6 μm s^−1^ and 100.0 ± 20.0 μm s^−1^, for the PC-3 and SK-BR-3 cancer cells, respectively) of a light image that could manipulate cells occurred at the voltage magnitude and frequency of 10 Vpp and 3 MHz, respectively. The above-mentioned cell manipulation velocity ranges were technically sufficient for various applications. Overall, this study fundamentally investigated the effect of ODEP electrical conditions on the cellular properties of cancer cells. The information obtained is crucially important for the utilization of ODEP-based cell manipulation in a microscale system for various applications.

## Supplementary Materials

The following are available online at https://www.mdpi.com/2079-6374/10/6/65/s1, Figure S1: Photograph of the overall experimental setup, Figure S2: Comparison of relative cell viability (%) of the PC-3 cancer cells treated with varied ODEP exposed times as indicated (electrical condition: 10 Vpp and 5 MHz; ODEP exposed time: 1~15 minutes) [NS: No significant difference (*p* > 0.05), * significant difference (*p* < 0.05)].
